# Case Report: Adrenal angiomatoid fibrous histiocytoma misdiagnosed as pheochromocytoma

**DOI:** 10.3389/fonc.2025.1578541

**Published:** 2025-10-06

**Authors:** Ziyou Yao, Yinqi Peng, Xianze Bi, Wenyuan Chen, Bo Wang, Jiange Zhang, Haipeng Huang

**Affiliations:** Department of Urology, The Second Affiliated Hospital of Guangxi Medical University, Nanning, Guangxi, China

**Keywords:** adrenal angiomatoid fibrous histiocytoma, case reports, soft tissue, surgical resection, laparoscopic adrenalectomy

## Abstract

**Background:**

Adrenal angiomatoid fibrous histiocytoma (AFH) is a rare soft tissue tumor that is frequently misdiagnosed preoperatively. This diagnostic challenge is compounded by its nonspecific clinical presentation and radiological features, which often overlap with more common adrenal neoplasms such as pheochromocytoma, adrenocortical carcinoma, and metastasis. This report describes a case of adrenal AFH that was successfully managed via retroperitoneal laparoscopic adrenalectomy.

**Case introduction:**

An 18-year-old male presented with a two-week history of recurrent abdominal pain and vomiting. Preoperative computed tomography angiography and urography suggested a pheochromocytoma. Pheochromocytoma was initially suspected based on preoperative computed tomography angiography and urography findings; however, postoperative pathological analysis confirmed the diagnosis as adrenal angiomatoid fibrous histiocytoma. There was no recurrence of adrenal angiomatoid fibrous histiocytoma during the follow-up of 10 months.

**Conclusions:**

Adrenal AFH is a rare tumor with a high propensity for misdiagnosis. It should be considered in the differential diagnosis of adrenal masses with imaging features suggestive of hemangioma. Surgical resection is the primary treatment, and the prognosis is generally favorable without the need for adjuvant radiotherapy or chemotherapy. Long-term surveillance is recommended due to its intermediate biological potential and documented risk of late recurrence.

## Background

Angiomatoid fibrous histiocytoma (AFH) is an exceptionally rare soft tissue tumor of intermediate (low-grade malignant) potential. First described by Enzinger in 1979 (1), it predominantly affects children and young adults. While the extremities and trunk are the most common sites, cases have been reported in various locations, including the brain, lungs, and retroperitoneum (2–4). The imaging features of adrenal AFH are nonspecific, making it difficult to distinguish from other adrenal tumors. This diagnostic ambiguity often leads to preoperative misdiagnosis, potentially impacting perioperative planning and patient counseling.

The management of adrenal tumors has been transformed by minimally invasive surgery (MIS), which is now the standard of care for most benign and select malignant lesions. Laparoscopic adrenalectomy, via either the transperitoneal or retroperitoneal approach, offers considerable advantages over open surgery, including reduced intraoperative blood loss, shorter hospital stay, less postoperative pain, and better cosmetic outcomes (5). The retroperitoneal approach, utilized in this case, allows direct access to the adrenal gland without entering the peritoneal cavity, potentially lowering the risk of visceral injury and promoting faster recovery (6). This approach is particularly advantageous for posteriorly located adrenal tumors, as it provides a shorter, more direct operative pathway. Although preoperative suspicion of pheochromocytoma mandates thorough perioperative preparation to avoid hemodynamic instability, MIS principles can be safely applied with appropriate surgical and anesthesiological expertise. The successful application of laparoscopic techniques in this case of a large (7.5 cm) AFH further underscores the versatility and safety of MIS for managing sizable adrenal masses of uncertain pathology, provided that oncological principles of complete resection with negative margins are strictly adhered to.

We report a case of a large adrenal AFH (75×51×37 mm) that was preoperatively misdiagnosed as a pheochromocytoma and successfully treated with laparoscopic adrenalectomy.

## Case study

On May 7, 2023, an 18-year-old male was admitted to the Second Affiliated Hospital of Guangxi Medical University for recurrent abdominal pain and vomiting lasting over two weeks. The abdominal pain was described as a dull, persistent ache in the right upper quadrant, without specific aggravating or relieving factors. The vomiting was non-bilious and occurred predominantly postprandially, suggesting a possible mass effect on adjacent gastrointestinal structures.

## Past medical history

The patient was previously healthy with an unremarkable medical history. There was no family history of endocrine disorders, neurocutaneous syndromes, or other inherited cancer predisposition syndromes that are commonly associated with adrenal pathologies. Physical examination on admission revealed a conscious patient with stable vital signs. No hypertension, chills, fever, dizziness, nausea, vomiting, urinary frequency, urgency, dysuria, hematuria, or pyuria was noted.

## Laboratory tests

Routine laboratory parameters were within normal limits. Detailed results of hematological, biochemical, coagulation, and tumor marker tests are summarized in **Table 1**.

**Table 1:** Laboratory test results on admission.

Test item	Result	Reference range
Hematology		
White blood cell count (×10^9^/L)	9.51	4.0-10.0
Red blood cell count (×10¹²/L)	4.62	4.3-5.8
Hemoglobin (g/L)	125.00	130-175
Platelet count (×10^9^/L)	331	125-350
C-reactive protein (mg/L)	30.33	0-6
Blood biochemistry		
Urea nitrogen (mmol/L)	4.41	2.9-8.2
Serum creatinine (μmol/L)	86	59-104
Uric acid (μmol/L)	434	208-428
Albumin (g/L)	44	40-55
Alkaline phosphatase (U/L)	103	45-125
Alanine aminotransferase (U/L)	16	9-50
Aspartate aminotransferase (U/L)	57	15-40
Potassium (K^+^, mmol/L)	4.50	3.5-5.3
Sodium (Na^+^, mmol/L)	139.0	137-145
Chloride (Cl^-^, mmol/L)	107.6	99-110
Calcium (Ca²^+^, mmol/L)	2.45	2.2-2.7
Magnesium (Mg²^+^, mmol/L)	0.80	0.75-1.05
Coagulation profile		
Prothrombin time (PT, S)	14.30	11.0-15.0
Activated partial thromboplastin time (APTT, S)	33.90	29.0-42.0
Fibrinogen (FIB, g/L)	4.05	2.0-4.0
Thrombin time (TT, S)	12.90	14.0-21.0
Tumor markers		
Carcinoembryonic antigen (ng/mL)	1.18	0-5.0
Alpha-fetoprotein (AFP, ng/mL)	0.91	0-7.0
Carbohydrate antigen 125 (CA125, U/mL)	13.00	0-35.0
Carbohydrate antigen 153 (CA153, U/mL)	6.80	0-25.0
Carbohydrate antigen 199 (CA199, U/mL)	0.00	0-37.0
Carbohydrate antigen 242 (CA242, U/mL)	0.035	0-20.0
Carbohydrate antigen 724 (CA724, U/mL)	2.60	0-6.9

Urinalysis and stool occult blood test were negative. Electrocardiogram and plain radiographs of the chest and abdomen showed no obvious abnormalities.

## Imaging studies

**Figure 1:**
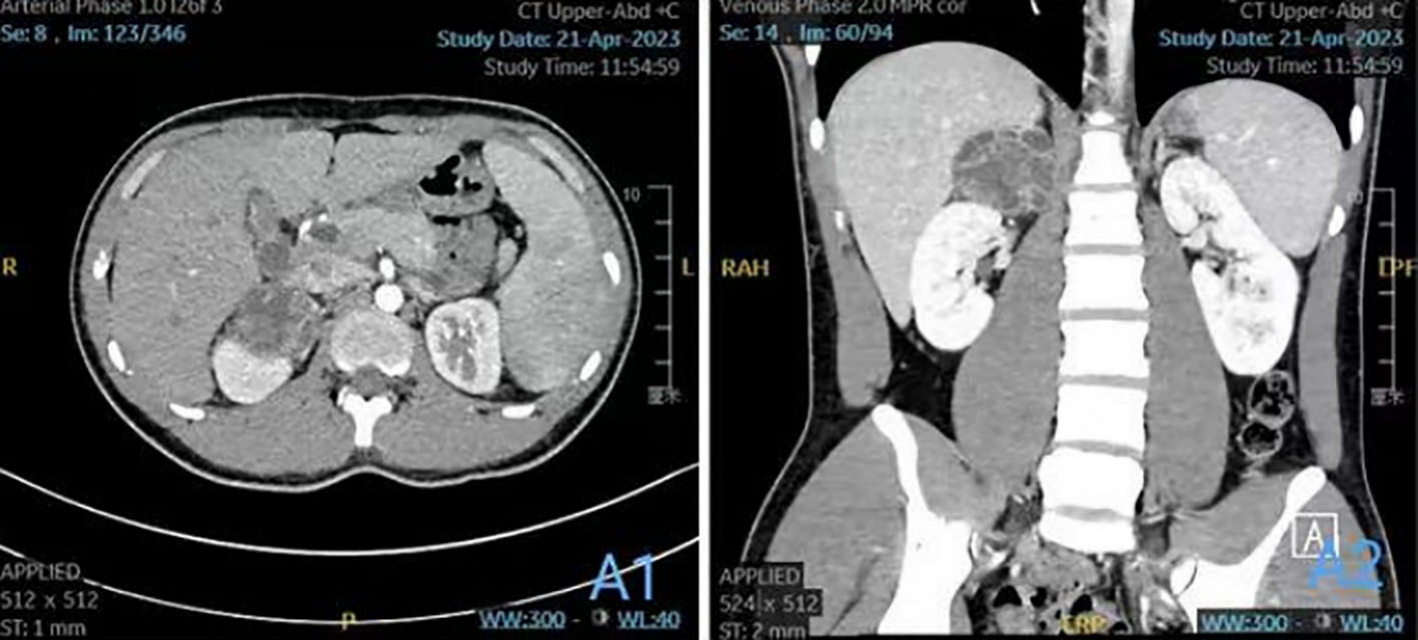
**(A1)** Plain and enhanced CT scan of the upper abdomen; **(A2)** coronal image.

Preoperative computed tomography angiography and urography (CTA+CTU) revealed a well-circumscribed, low-density mass (approximately 5.7×5.1×4.5 cm) in the right adrenal gland. The solid components showed enhancement on contrast administration (**Figures 1A1, A2**). The enhancement pattern was heterogeneous, with areas of avid contrast uptake in the arterial phase and washout in the delayed phase, features that can be associated with both hypervascular tumors like pheochromocytoma and certain soft tissue sarcomas. The liver, gallbladder, spleen, pancreas, and kidneys appeared normal in size, shape, and attenuation, with no abnormal enhancement. The abdominal bowel was normally distributed without dilation or effusion. No definite masses, enlarged lymph nodes (hepatic hilum or para-aortic), or peritoneal effusion were observed. The initial differential diagnosis included a left adrenal solid-cystic mass, with considerations of pheochromocytoma or ganglioneuroma.

## Treatment

The patient underwent retroperitoneal laparoscopic adrenalectomy. Preoperative management included oral phenoxybenzamine hydrochloride (10 mg twice daily for one week) and volume expansion via infusion two days prior to surgery to minimize intraoperative blood pressure fluctuations. This preoperative α-blockade was initiated empirically due to the high radiological suspicion for pheochromocytoma, highlighting the critical importance of preoperative preparation for the most dangerous potential diagnosis, even in the absence of biochemical confirmation. The procedure lasted 160 minutes and was performed under strict aseptic principles. Intraoperative anesthesia monitoring indicated stable hemodynamics during tumor manipulation. Following surgery, the patient was transferred to the ICU for continuous monitoring of vital signs over a 24-hour period. Once stability was confirmed, the patient was moved to the general ward for regular observation and was subsequently discharged 2 days postoperatively. The extended hospitalization was primarily due to cautious postoperative monitoring and recovery from the laparoscopic procedure, ensuring no delayed hemodynamic complications occurred.

## Follow-up

The patient recovered well from the surgery without any immediate postoperative complications. He was scheduled for regular outpatient follow-up appointments. Physical examination and abdominal computed tomography (CT) scans were performed at 3, 6, and 12 months postoperatively. At the time of writing this report (10 months after surgery), the patient remained asymptomatic with no clinical or radiological evidence of local recurrence or distant metastasis. Given the intermediate malignant potential of AFH, the follow-up protocol will be extended beyond the first year. We plan to continue annual clinical examination and cross-sectional imaging (CT or MRI) for at least 5 years, in accordance with guidelines for managing intermediate-grade soft tissue sarcomas, to vigilantly monitor for any signs of late recurrence or metastasis.

## Pathological results

**Figure 2:**
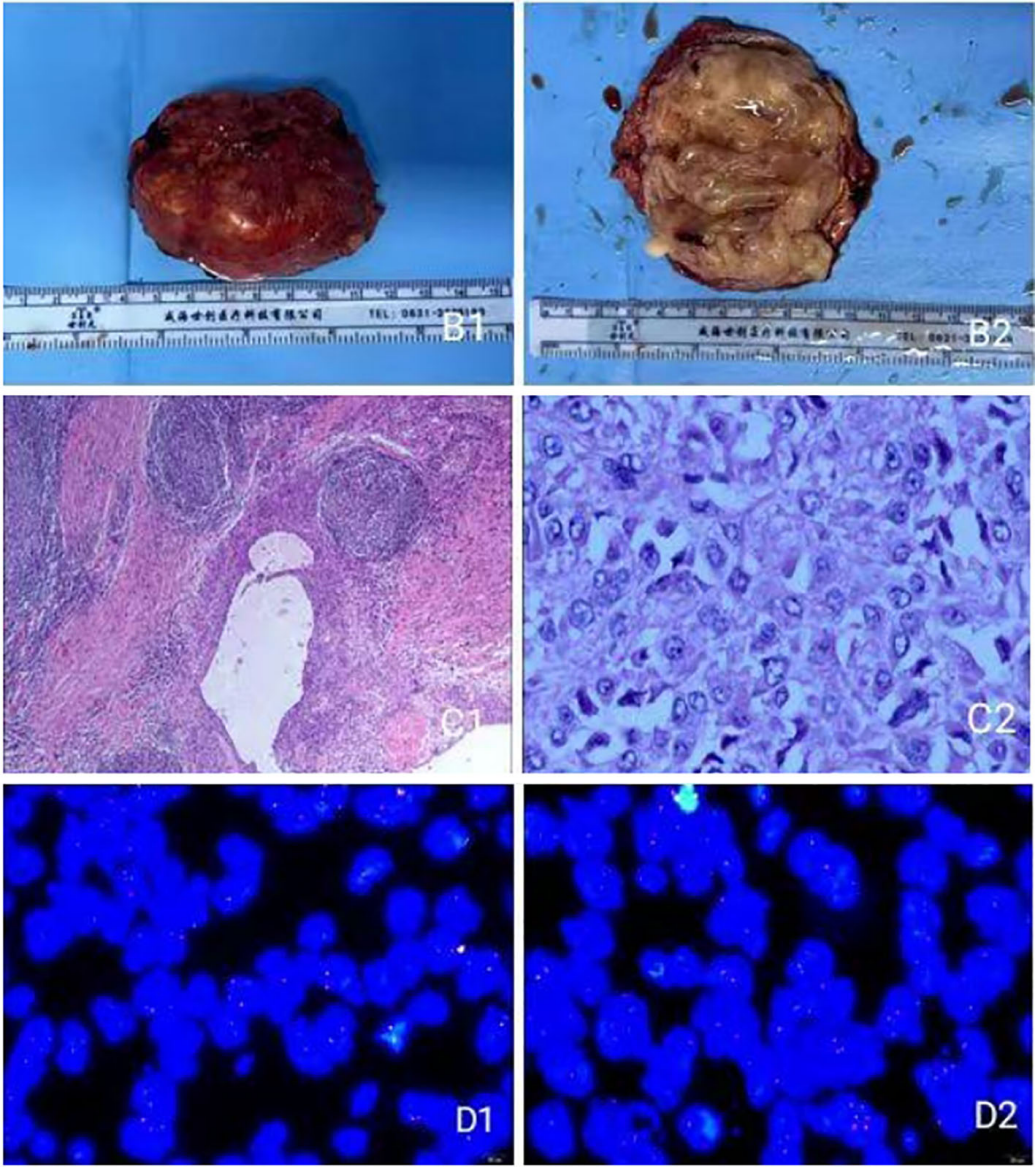
**(B1)** Gross view of the tumor; **(B2)** Cross-sectional view of the tumor; **(C1**, **C2)** under light microscope; **(D1**, **D2)** FISH assay display.

**Macroscopic examination:** The tumor measured 7.5 × 5.1 × 3.7 cm. It was a grayish-brown, well-encapsulated mass. The cut surface appeared brownish with solid and cystic areas of medium texture. Irregular, slit-like cystic spaces of varying sizes containing yellow-brown fluid were observed. The cyst wall was irregular with small white-yellow nodules (**Figures 2B1, B2**). A portion of the resected adrenal gland was identified. The gross findings were highly characteristic of AFH, featuring a combination of solid tumor nodules and blood-filled cystic spaces simulating a vascular neoplasm, which explains its historical nomenclature.

**Microscopic examination:** The tumor was well-circumscribed by a thick fibrous pseudocapsule infiltrated by lymphocytes and plasma cells, forming a “peripheral cuff” that merged with the pseudocapsule. Tumor cells were arranged in solid sheets or nodules with focal storiform patterning (**Figures 2C1, C2**). The tumor cells were predominantly ovoid to spindle-shaped, with bland nuclear features and low mitotic activity. Foci of hemosiderin deposition were noted within the pseudocapsule and the tumor stroma, a common finding attributable to previous intratumoral hemorrhage.

**Immunohistochemical results:** Tumor cells were positive for CK (Pan), Vimentin, EMA, CD99, Desmin, CD68 (focal), Melan A (weak), ALK (focal), SMA, FLI1, and CD34. They were negative for CgA, Syn, S-100, Inhibin-α, STAT6, CD31, ERG, HMB45, CD117, CD21, CD35, and GFAP. The Ki-67 proliferation index was 5% in hotspots, with small foci positive for CD56.

**EWSR1 FISH analysis (** **Figures 2D1, D2** **):** Analysis of 100 tumor cells revealed the following signal patterns: 2F: 11%; 1F: 19%; nF: 2%; nGnRnF: 6%; 1F1R: 1%; 1F1G: 1%; nRnG: 26%; 1G1R1F: 34%. (G: green signal; R: red signal; F: yellow fusion signal). Conclusion: Positive for EWSR1 gene rearrangement.

## Discussion

AFH is a rare soft tissue tumor of intermediate (borderline) biological behavior. Initially termed “angiomatoid malignant fibrous histiocytoma” by Enzinger (1), it has been reclassified by the WHO due to its generally indolent clinical course and favorable prognosis, a point illustrated by our patient who remained asymptomatic and recurrence-free 10 months after surgical resection. This evolution in nomenclature reflects a better understanding of its biological potential, which is significantly less aggressive than that of true malignant fibrous histiocytoma (undifferentiated pleomorphic sarcoma).

The principal diagnostic challenge of adrenal AFH lies in its nonspecific preoperative presentation. As evidenced in our case, its imaging features can closely mimic other adrenal tumors, particularly pheochromocytoma. This initial misdiagnosis, however, dictated a critical and meticulous perioperative management strategy. Adhering to protocols for pheochromocytoma, we implemented preoperative α-adrenergic blockade and volume expansion. This approach was essential to mitigate the potential for catastrophic hemodynamic instability during manipulation of a suspected catecholamine-secreting tumor (7, 8), underscoring the importance of preparing for the worst-case scenario despite the eventual benign diagnosis. This case exemplifies a fundamental principle in adrenal surgery: the radiographic appearance, especially for large or hypervascular masses, must often take precedence over inconclusive biochemistry to ensure patient safety, erring on the side of caution.

The definitive diagnosis of AFH relies on a triad of histological, immunohistochemical, and molecular findings. Histopathologically, our case exhibited classic features: a well-circumscribed tumor with a thick fibrous pseudocapsule demonstrating a prominent lymphoplasmacytic infiltrate (“peripheral cuff”), pseudoangiomatoid hemorrhagic spaces, and proliferations of spindle cells in solid sheets with focal storiform architecture. Immunohistochemically, the tumor cells were positive for vimentin, CD68, EMA, and desmin, a profile highly suggestive of AFH. The expression of desmin, while not universal, is a particularly valuable clue, as it is uncommon in many other morphological mimics. This constellation of features necessitates differentiation from several morphologic mimics, including (1) fibrous histiocytoma (lacks desmin expression and EWSR1 rearrangement), (2) metastatic tumor to a lymph node (possesses true nodal architecture like marginal sinuses), (3) infantile fibrosarcoma (harbors ETV6-NTRK3 fusion), and (4) hematoma or hemangioma (shows positivity for specific vascular markers like CD31 and CD34, which were negative in our case) (9–12).

The diagnosis was conclusively confirmed by molecular testing. Fluorescence *in situ* hybridization (FISH) analysis revealed a disruption of the EWSR1 gene locus at 22q12, a genetic aberration reported in a majority of AFH cases (4, 13). This case exhibited a complex pattern of signal splits (e.g., 1F1R: 1%, 1G1R1F: 34%), confirming the genetic signature that underpins AFH pathogenesis, most commonly through fusions such as EWSR1-CREB1 or EWSR1-ATF1 (4). The integration of FISH testing is increasingly becoming an indispensable tool for resolving diagnostically challenging cases. In fact, for tumors with atypical presentation or morphology, molecular confirmation has transitioned from a helpful ancillary test to a near-mandatory component of the diagnostic workup.

Given its intermediate malignant potential, the cornerstone of AFH management is complete surgical excision. Extensive local resection is recommended, as recurrence is occasionally associated with positive margins, deep location, or incomplete resection (14, 15). The role of adjuvant radiotherapy or chemotherapy is reserved for unresectable or recurrent disease (16). In the present case, the patient was successfully treated with retroperitoneal laparoscopic adrenalectomy alone. He has been under close surveillance with clinical and radiological examinations, and at 10 months postoperatively, there is no evidence of recurrence or metastasis. This favorable early outcome aligns with the generally good prognosis of this tumor entity; however, given its known potential for late recurrence, long-term follow-up remains imperative.

Despite the valuable insights provided by this case, our study has several limitations that should be acknowledged. Firstly, this is a single-case report, which inherently limits the generalizability of our findings. The conclusions drawn are specific to this patient’s presentation and outcome. Secondly, and most notably, the follow-up period of 10 months is relatively short for a tumor of intermediate biological behavior like AFH. While the patient remains disease-free, late recurrence, although uncommon, has been reported in AFH. Therefore, a longer-term follow-up is essential to definitively confirm the excellent prognosis in this particular case. Future multi-institutional collaborations and long-term registries are needed to establish more robust evidence-based guidelines for the management and surveillance of this rare entity, particularly in its unusual locations like the adrenal gland.

In summary, this case highlights adrenal AFH as a great mimicker in the adrenal gland. It reinforces the necessity of including rare mesenchymal tumors in the differential diagnosis of adrenal masses, especially in young adults. A high index of suspicion, coupled with meticulous histopathological examination, judicious application of immunohistochemistry, and confirmatory molecular testing, is paramount for an accurate diagnosis. Lastly, it demonstrates that laparoscopic resection is a feasible and effective treatment option, provided that cautious perioperative management is employed when the diagnosis is uncertain.

## Data Availability

The original contributions presented in the study are included in the article/supplementary material. Further inquiries can be directed to the corresponding author.
